# Hydrogel Encapsulation: Taking the Therapy of Mesenchymal Stem Cells and Their Derived Secretome to the Next Level

**DOI:** 10.3389/fbioe.2022.859927

**Published:** 2022-04-01

**Authors:** Yuling Huang, Xin Li, Lina Yang

**Affiliations:** ^1^ Departments of Geriatrics, First Affiliated Hospital of China Medical University, Shenyang, China; ^2^ Departments of Infectious Disease, First Affiliated Hospital of China Medical University, Shenyang, China

**Keywords:** hydrogel, extracellular vesicles, biomaterials, secretome, mesenchymal stem cells

## Abstract

Biomaterials have long been the focus of research and hydrogels are representatives thereof. Hydrogels have attracted much attention in the medical sciences, especially as a candidate drug-carrier. Mesenchymal stem cells (MSC) and MSC-derived secretome are a promising therapeutic method, owing to the intrinsic therapeutic properties thereof. The low cell retention and poor survival rate of MSCs make further research difficult, which is a problem that hydrogel encapsulation largely solved. In this review, safety and feasibility of hydrogel-encapsulated MSCs, the improvement of the survival, retention, and targeting, and the enhancement of their therapeutic effect by hydrogels were studied. The status of the hydrogel-encapsulated MSC secretome was also discussed.

## 1 Introduction

The study of biomaterials has been advancing rapidly in recent years. On the one hand, biomaterials can fill and repair tissue defects directly as accessible grafts, for example, calcium phosphate materials have been studied widely in the treatment of bone defects (Humbert et al., 2019). On the other hand, biomaterials enhance the effects of other therapies by various methods, such as drug delivery, targeted injury, and controlled release. Of those biomaterials, hydrogels have attracted attention in drug-delivery investigations due to their outstanding biocompatibility, degradability, and processability. There are various preparation methods for hydrogels, including covalent bonding, photopolymerization, thermo-gelation hydrogels, cryo-gelation, and other non-covalent bonding (Huang et al., 2017a). According to the origin of the gel precursor molecules, hydrogels can be divided into natural and synthetic hydrogels: the former hydrogels demonstrate better biocompatibility; while the latter has better stability (Salinas and Anseth, 2009). Additionally, based on application purpose, strong machinability enables hydrogels to be formed into different shapes and sizes, such as fibers, microbeads, or nanoparticles (Pishavar et al., 2021).

MSCs, multipotential stem cells, are derived from various tissues, including bone marrow (BM-MSCs), adipose (AD-MSCs), umbilical cord (UC-MSCs), peripheral blood, etc. According to “Minimal criteria that define multipotent mesenchymal stromal cells,” MSCs adhere to plastics under standard culture conditions; CD105, CD73 and CD90 are specific surface markers; through *in vitro* induction, MSCs can differentiate into bone, adipose, and cartilage (Dominici et al., 2006). Early studies also generally suggested that differentiation is the main mechanism of MSC therapy (Lange et al., 2005). With further exploration, secretomes such as soluble trophic factors and extracellular vesicles (EVs) play a more important role in the treatment of MSCs. The MSC secretome includes soluble trophic factors (secreting growth factors, chemokines, cytokines, etc.) and EVs. The sac-like structures formed by EVs can encapsulate specific factors (DNA, RNA, protein, and amino acids metabolites) secreted by mother cells to transmit information in a stable manner *in vivo* and vitro (Kalluri and LeBleu, 2020). Numerous preclinical studies confirmed the benefits of MSCs and their secreted factors. For example, UC-MSC administration improved the outcomes of patients with severe COVID-19 pneumonia without any infusion reaction (Guo et al., 2020). Mesenchymal stem cell-derived extracellular vesicles (MSC-EVs) attenuated renal inflammation and fibrosis in pigs with metabolic syndrome and renal artery stenosis (Eirin et al., 2017).

**FIGURE 1:**
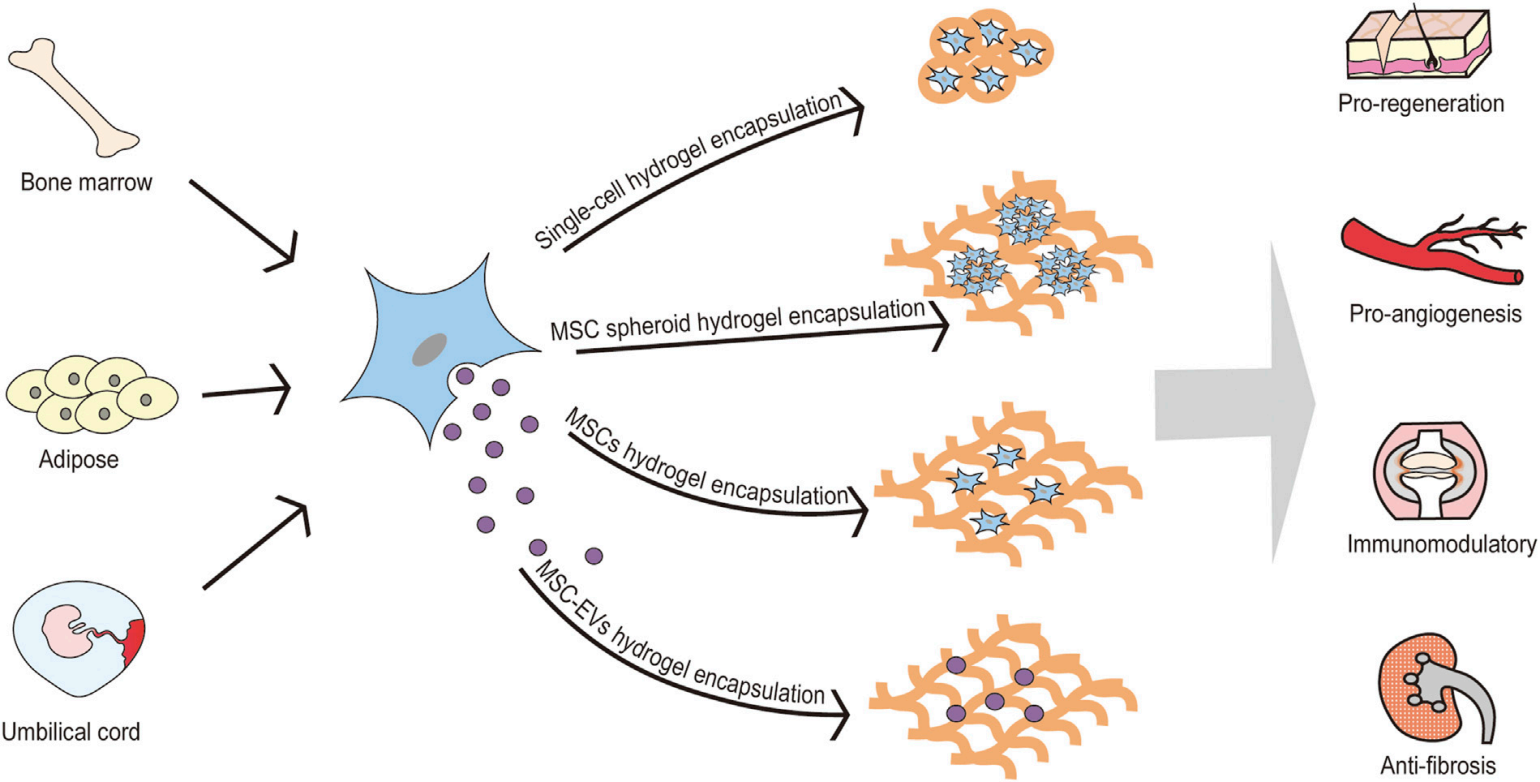
Hydrogel encapsulation may bring the treatment of MSCs and their secretome to the next level. MSCs are derived from various tissues, including bone marrow, adipose, umbilical cord, peripheral blood, etc. MSCs can be encapsulated by hydrogel in various formation (single-cell hydrogel encapsulation, MSC spheroid hydrogel encapsulation, and MSCs hydrogel encapsulation). Also, MSC-EVs can be encapsulated by hydrogel for pro-regenerative, pro-angiogenic, immunomodulation and anti-fibrotic effects. MSC: mesenchymal stem cell; MSC-EVs: mesenchymal stem cell-derived extracellular vesicles.

Infusion of MSCs and their derived secretome, as an emerging therapeutic modality, which can be proregenerate, proangiogenisis, immunomodulate, anti-inflammatory, anti-fibrotic, etc. has been recognized by experts in many diseases. Clinically, MSCs have been approved for the treatment of Crohn’s complicated intestinal fistula and graft-versus-host disease. However, the non-targeted distribution of MSCs and their derived secretomes, low tissue retention, and high metabolic rate greatly limit their applications. Hydrogel encapsulation can solve the above problems and provide a new drug route for MSCs. More interestingly, incorporation of various bioactive factors can improve directional delivery and promote specific behavior of MSCs. Therefore, hydrogel encapsulation may bring the treatment of MSCs and their secretome to the next level (Figure 1). The manuscript will be generally introduced from two aspects: hydrogels encapsulate MSC and hydrogels encapsulate MSC secretome: the former includes premise, mechanism, and advance of hydrogels-encapsulated MSCs; the latter is composed according to the classification and components of MSC secretome.

## 2 Hydrogels Encapsulate Mesenchymal Stem Cells

Biomaterials combined with MSCs were initially mainly used for tissue regeneration, especially bone regeneration. Researchers found that the addition of MSCs can significantly promote regeneration compared with the filling of defective bone tissue with biomaterials (De Kok et al., 2003). Later, the same researchers proposed that injecting the hydrogel could help stimulate bone marrow to produce stem cells to grow new cartilage in cartilage repair surgery (Huang et al., 2010). Subsequently, hydrogels have been widely recognized as a high water-absorbing and high water-holding material to load medicines in drug delivery applications. It is noteworthy that the focus of the present research is to encapsulate MSCs with hydrogels to achieve targeted delivery, increase the retention at the injury site, and enhance the function of MSCs. This is different from the addition of MSCs when the biomaterial fills the defective tissue. A wide variety of hydrogels are used to encapsulate MSCs. The main natural polymer hydrogels are comprised of alginic acid (Ho et al., 2016), hyaluronic acid (Liu et al., 2019), chitosan (Boido et al., 2019) and collagen (Huang et al., 2017b), etc. Synthetic hydrophilic polymers include polyethylene glycol (PEG) (Sylvester et al., 2021) and acrylic acid (Qiu et al., 2011). In addition, based on a single hydrogel, mixing other hydrogels and incorporating some bioactive factors can guide MSCs to exert more specific functions. Hydrogel-encapsulated MSCs are widely used, and existing studies involve various tissues and organs: bone, cartilage, heart, kidney, skin, spinal cord, etc.

### 2.1 Safety and Feasibility of Hydrogels-Encapsulated Mesenchymal Stem Cells

The safety and feasibility of hydrogel-encapsulated MSCs are the prerequisites for the clinical translation of this therapy. On the one hand, whether MSCs can successfully perform their therapeutic functions in hydrogels is very important. Wu et al. found that self-assembled supramolecular hydrogel-encapsulated MSCs could maintain cell morphology and viability, which demonstrate the biocompatibility and non-cytotoxicity of the hydrogels (Wu et al., 2008). In the chitosan-based hydrogel developed by Boido et al., the viability and paracrine activity of MSCs were not affected by the hydrogel, and the encapsulated MSCs could release MSC vesicles and maintain their antioxidant function (Boido et al., 2019). Papa et al. found that MSCs in arginine-glycine-aspartate (RGD) -extracellular matrix hydrogel scaffolds could maintain cellular structure *in situ* and gradually release CCL2 chemokine, which can promote functional recovery from spinal cord injury (SCI) (Papa et al., 2018). The study of Bussche et al. showed that the microencapsulation of MSCs did not interfere with the release of bioactive factors (Bussche et al., 2015). Furthermore, co-gelation of acellular vascular matrix and collagen enhanced the survival and paracrine effects of MSCs in the injured kidney (Huang et al., 2017b). However, some studies pointed out that the 3D microenvironment hinders the trans-differentiation of MSCs and the production of secreted factors in the onerous microenvironment of myocardial infarction (MI) (Sylvester et al., 2021).

On the other hand, the biocompatibility of hydrogels as allografts is worth investigating. Yuan et al. studied and compared the changes in immunological properties associated with different scaffolds and found that MSC-hydrogel structures caused rather low proliferation of allogeneic lymphocytes, especially when prepared from higher concentrations of collagen in the hydrogel (Yuan et al., 2011). Also, attenuating innate immune responses could be achieved by modifying hydrogels. Ghanta et al. used immune-evasive and small-molecule-modified alginate encapsulation to enable MSCs to persist and localize on the heart, and then to improve cardiac function after acute MI (Ghanta et al., 2020). Similarly, Alvarado-Velez et al. engineered an agarose hydrogel that releases Fas ligand, a protein that induces apoptosis in cytotoxic CD8^+^ T cells, to increase MSC survival rate of allogeneic transplantation near the injury site (Alvarado-Velez et al., 2021). Moreover, in the clinical study of He et al., intramyocardial injection of UC-MSC-loaded collagen hydrogel showed no serious adverse reactions, proving that the use of collagen hydrogel for cell delivery is safe and feasible (He et al., 2020).

### 2.2 Hydrogel Encapsulation Improves Mesenchymal Stem Cells Survival, Retention, and Targeting

#### 2.2.1 Improving Mesenchymal Stem Cells Survival

Before encapsulation, several conditions affected the viability of MSCs. Chen et al. proposed hydrogel-induced BM-MSCs under conditions of cell density (<2 × 10^7^ cells/mL), DMSO concentration (<0.5%), and needle gauge (25G or 27G). The survival rate could be maintained above 82% (Chen et al., 2017). Osama et al. proposed a different view: MSCs injected with a 30G needle showed significantly better viability when the hydrogel was in the pre- and post-gel state (Osama et al., 2018). Additionally, the different kinds of hydrogel encapsulation can play different effects. For example, single-cell hydrogel-encapsulated MSCs could interact with the cell-matrix via the activation of ERK/MAPK signaling cascade, thereby increasing cell viability (Karoubi et al., 2009). Isolated MSCs/secretome hydrogel encapsulation is applied in most experiments. Compared with isolated MSCs, MSC spheroids exhibit higher therapeutic potential in various aspects, improving cell survival, anti-inflammatory and angiogenic potential (Ho et al., 2016).

Adding some chemicals or bioactive factors to the hydrogel also promoted the viability of MSCs (Table 1). Cellulose nanocrystal-enhanced collagen-based nanocomposite hydrogels protected cells during injection (Zhang et al., 2020a). In the hypoxic environment of MI, graphene oxide added to alginate composite microgels protected MSCs from the harsh hypoxic environment (Choe et al., 2019). Compared to chemical modification, bioactive factors appeared to have better biocompatibility. Superoxide dismutase also protected MSCs encapsulated in hydrogels from superoxide attack (Li et al., 2009). The platelet-rich plasma gel provided a favorable survival environment for MSCs, and the optimal platelet concentration was 1 × 10^6^ platelets/μl (Jalowiec et al., 2016). Fibrin hydrogels that were loaded with platelet-derived growth factor BB doubled the survival rate of MSC spheroids, indicating that platelet-derived growth factor BB could serve as a biochemical cue to promote the survival of MSC spheroids *in vivo* (Zhao et al., 2020). Heparan sulfate mimetic addition to hydrogels restored extracellular matrix network and enhanced growth factor bioactivity, increasing cell engraftment and cell survival (Moussa et al., 2019). In addition, miR-21 was retained in collagen hydrogels, targeted to activate downstream therapeutic cytokines, and significantly enhanced the anti-apoptotic capacity of MSCs (Zhang et al., 2017).

**TABLE 1:** Some chemicals or bioactive factors in hydrogel promoting the viability of MSCs.

Factor	Type of hydrogels	MSC origin	Characteristic	Efficacy	References
Cellulose Nanocrystal	Collagen-Based Nanocomposite Hydrogel	BM-MSCs	fast shear thinning, self-healing and improved elastic modulus	high cell viability after extrusion *in vitro*, improved implant integrity and higher cell retention	Zhang et al. (2020a)
Reduced graphene oxide	Reduced graphene oxide	UC-MSCs	Anti-oxidant activity	higher cell viability and cardiac maturation	Choe et al. (2019)
ROS	Collagen biocomposite	BM-MSCs	Anti-oxidant activity	suppressed superoxide penetration into the hydrogel and cell membrane and stimulating MSC growth	Li et al. (2009)
Platelet-rich plasma	chitosan, batroxobin, thrombin, calcium chloride, or a combination of the latter two	BM-MSCs	promoting growth factor and inflammatory proteins release	the highest cell viability and DNA content found in PRP-gels with 1×10^6^ platelets/mL	Jalowiec et al. (2016)
PDGF-BB	Aptamer-functionalized fibrin hydrogel	MSC spheroids	Inhibiting the apoptosis and promoting the proliferation	promoted the survival of MSC spheroids	Zhao et al. (2020)
Heparan sulfate mimetics	Si-HPMC hydrogel	AD-MSCs	Restore the extracellular matrix network and enhance the biological activity of growth factors	increased cell engraftment and cell survival, and improved the therapeutic benefit	Moussa et al. (2019)
miR-21	Collagen hydrogel	AD-MSCs	interfering the expressions of apoptotic related proteins	protect MSCs from ROS-induced cellular dysfunction	Zhang et al. (2017)

Adding some chemicals or bioactive factors to the hydrogel also promoted the viability of MSCs. Compared to chemical modification, bioactive factors appeared to have better biocompatibility. MSC: mesenchymal stem cell; ROS: reactive oxygen species; BM-MSCs: MSCs derived from bone marrow; UC-MSCs: MSCs derived from umbilical cord; AD-MSCs: MSCs derived from adipose.

#### 2.2.2 Improving Mesenchymal Stem Cells Retention, and Targeting

Obviously, hydrogel encapsulation can improve MSC retention, which is closely related with different kinds of hydrogel. The degradation rate of the hydrogel was found to determine the cell delivery, retention, and controlled release to a certain extent (Qiu et al., 2011). In a femoral defect model, Hoffman et al. controlled longitudinal localization of MSCs on the surface of allografts by altering hydrogel degradation kinetics (Hoffman et al., 2014).

Hydrogel encapsulation can improve efficient distribution and targeting of MSCs. The current research mainly achieves target of MSC through direct contact. For example, covering the surface of injured skin can limit the local spread of MSCs (Zhou et al., 2019a). Intramyocardial injection of MSC-loaded collagen hydrogel can improve distribution of MSCs in heart (He et al., 2020). Also, modifying hydrogel is a feasible method, but the research has not applied in hydrogel encapsulation. For example, by the coating of lipid-conjugated heparin, AD-MSCs without hydrogel encapsulation can target the damaged liver with enhanced delivery and longer retention (Hwang et al., 2019).

### 2.3 Hydrogel Encapsulation Guide Mesenchymal Stem Cells to Perform Specific Functions Using Tailored Biochemical and Biophysical Cues

MSCs have the properties of tissue repair ability and immune regulation ability. Hydrogel encapsulation enhances the therapeutic capacity of MSCs by increasing survival, retention, and targeting. The therapeutic efficacy of hydrogel-loaded MSCs has been demonstrated in many diseases, for example, the function of gel-encapsulated MSCs has been continuously explored in bone and cartilage regeneration, wound repair, MI, and other studies.

#### 2.3.1 Tissue Repair

Angiogenesis plays an irreplaceable role in the process of tissue repair. Glycine-histidine-lysine-modified alginate hydrogels could upregulate the ability of MSCs to secrete pro-angiogenic factors (Jose et al., 2014). Intramuscular injection of MSCs within a PEGylated fibrin gel matrix was found to promote the formation of mature blood vessels in a model of hind limb ischemia (Ricles et al., 2016). Also, a novel hydrogel composed of pooled human platelet lysates could localize ischemic tissue to promote angiogenesis (Robinson et al., 2016).

MI is a common ischemic disease. Gao et al. used BG/γ-PGA/CS hydrogel to activate the interaction between MSCs and cell matrix, resulting in decreased apoptosis and enhanced angiogenesis, improved cardiac function, and attenuated cardiac remodeling (Gao et al., 2020). In addition, epicardial placement of MSCs-loaded POx hydrogels also improved neovascularization in rats with MI (You et al., 2021).

In wound repair, the pro-angiogenic effect of MSCs is crucial. Pharmacological pre-treatment of MSCs with the natural potent antioxidant was reported to increase the amount of pro-angiogenic cytokines and the speed of wound closure (Touani et al., 2021). The pore size of the hydrogel scaffolds can also act as a regulator of the paracrine effects of MSC angiogenesis, with medium-pore-size scaffolds having the highest paracrine effects of angiogenic cytokines (Qazi et al., 2019). MSC spheroids are currently being studied more, and compared with isolated MSCs, MSC spheroids exhibit higher therapeutic potential in various aspects, improving cell survival, anti-inflammatory and angiogenic potential (Ho et al., 2016). Especially in the promotion of wound healing, multiple studies showed that hydrogel-encapsulated MSC spheroids have faster wound closure and more obvious angiogenesis (Murphy et al., 2017; Yang et al., 2020a). For contaminated wounds, adding antibiotics such as minocycline (Guerra et al., 2017) or FMZC to the base hydrogel encapsulating MSCs was able to reduce bacterial bioburden and promote wound repair (Rafie and Meshkini, 2021).

#### 2.3.2 Immunomodulatory and Anti-Fibrotic

In myocardial ischemia/reperfusion injury, MSCs encapsulated in a hydrogel carrier mediate the conversion of AMP to adenosine by CD73, exerting a powerful anti-inflammatory effect (Shin et al., 2020). In osteoarthritis, Gómez-Aristizábal et al. found that hyaluronic acid binding to MSCs has an additive effect on MSC-mediated immune modulation (Gómez-Aristizábal et al., 2016). Sodium alginate microencapsulation could also modulate MSC paracrine function and enhance the therapeutic effect of MSCs in OA (McKinney et al., 2021). In injured vocal cords, MSCs + hyaluronic acid hydrogel exerted an equivalent inhibitory effect on inflammation *in vivo* (Hertegård et al., 2019). In colonic radiation-induced injury, MSCs in silylated hydroxypropylmethylcellulose hydrogel were capable of secreting trophic factors and responded to the inflammatory milieu (Moussa et al., 2017). In addition to immune regulation, anti-fibrosis is also an important function of MSCs. Gelatin-microcryogel-loaded MSCs protected 5/6 nephrectomized kidneys through anti-inflammatory and anti-fibrotic effects (Geng et al., 2019). In a radiation therapy-induced esophageal fibrosis model, Kim et al. used catechol-functionalized hyaluronic acid hydrogel-encapsulated MSC spheroids to improve esophageal injury (Kim et al., 2021).

## 3 Hydrogels Encapsulate Mesenchymal Stem Cells Secretome

According to the current study, MSCs mainly exert their therapeutic effects through paracrine. Compared with MSCs, MSC secretome has more advantages: 1. Cell-free treatment reduces the requirement for MSCs, resulting in a large amount of storage and transportation logistics; 2. MSC secretome shows a smaller risk of embolism; 3. MSC secretome avoids the potential of MSC tumorigenicity. Therefore, hydrogel-encapsulated MSC secretome in treating diseases is also a key research topic (Table 2).

**TABLE 2:** Hydrogel-encapsulated MSC secretome in treating diseases.

Type of hydrogel	MSC origin	Type of secretome	Administration	Mechanism	Application	References
Hyaluronic acid hydrogel	BMMSCs	Non-specific	Intrauterine administration	Restored endometrial morphology and function	Asherman’s Syndrome	Liu et al. (2019)
Composed of COLI and LMWHA or COLI and PEG	ADMSCs	Non-specific	*In vitro* study	Counteract 6-OHDA toxicity with upregulation of the antioxidant enzyme sirtuin 3	Parkinson’s disease	Chierchia et al. (2020)
GelMA PEGDA hybrid hydrogels	BMMSCs	Conditioned media	*In vitro* study	Promoted proliferative and migratory activities of hyperglycemic fibroblasts	Diabetic or chronic wounds	Sears et al. (2021)
Collagen hydrogel	ADMSCs	Non-specific	*In vitro* study	Increased proliferation of skin-origin cells and improved angiogenic properties of endothelial cells	Chronic wounds	Kraskiewicz et al. (2021)
Chitosan/collagen/β-glycerophosphate thermosensitive hydrogel	UCMSCs	Conditioned media	Covering the wounds	Limited the area of inflammation, enhanced reepithelialization, promoted the formation of granulation tissue, and attenuated the formation of fibrotic and hypertrophic scar tissue	Burn wounds	Zhou et al. (2019a)
Self-assembling peptide nanofiber hydrogel	BMMSCs	EVs	Renal capsule injection	Reduced tubular cell apoptosis, pro-inflammatory cytokine expression, and macrophage infiltration	Renal ischemia-reperfusion injury	Zhou et al. (2019b)
RGD hydrogel	hP-MSCs	EVs	kidney cortex injection	Facilitated MSC derived let-7a-5p-containing-EVs, improved reparative potential against AKI	AKI	Zhang et al. (2020b)
PEG hydrogel	ES-MSCs	EVs	Systemic administration	Improved the anti-fibrosis, anti-inflammation, anti-apoptosis and regenerative effects of EVs	Chronic liver fibrosis	Mardpour et al. (2019)
Chitosan hydrogel	hP-MSCs	EVs	Subcutaneous injection	Delayed the skin aging processes by ameliorating the function of aging DFLs	Aging skin	Zhao et al. (2021)
GelMA hydrogel	BMMSCs	EVs	Sprayed onto the surface of the heart	Alleviated apoptosis and promote angiomyogenesis	MI	Tang et al. (2021)
Pluronic F127 hydrogel	UCMSCs	Exosomes	Covering the wounds	Enhanced regeneration of granulation tissue and upregulated expression of VEGF and TGFβ-1	Chronic wounds	Yang et al. (2020b)
Peptide-modified adhesive hydrogel	hP-MSCs	Exosomes	Intravenous injection	Elicited significant nerve recovery and urinary tissue preservation by effectively mitigating inflammation and oxidation	Spinal cord injury	Li et al. (2020)
Chitosan hydrogel	hP-MSCs	Exosomes	Intramuscular injection	Improved survival and angiogenesis of endothelial cells and accelerated the recovery of ischemic hindlimbs	Hindlimb ischemia	Zhang et al. (2018)
Diels–Alder crosslinked hyaluronic acid/PEG hydrogel	IMSCs	sEVs	Intraarticular injection	Improved the bioavailability and therapeutic efficacy of MSC-sEVs for OA improvement	Osteoarthritis	Yang et al. (2021)
Sodium alginate hydrogel	BMMSCs	sEVs	Intramyocardial injection	Promoted angiogenesis, reduced cardiac apoptosis and fibrosis, enhanced scar thickness, and eventually improved cardiac function	MI	Lv et al. (2019)

Multiple experiments have shown that hydrogels can continuously release MSC secretome, and effectively exert pro-regenerative, pro-angiogenic, and anti-fibrotic effects. MSC: mesenchymal stem cell; BM-MSCs: MSCs derived from bone marrow; UC-MSCs: MSCs derived from umbilical cord; AD-MSCs: MSCs derived from adipose; hP-MSCs: MSCs derived from human placenta; IMSCs: induced MSCs; COLI: collagen type I; LMWHA: low molecular weight hyaluronic acid; RGD: arginine-glycine-aspartate; PEG: polyethylene glycol; MI: myocardial infarction.

Just like MSCs, multiple experiments have shown that hydrogels can continuously release MSC secretome, and effectively exert pro-regenerative, pro-angiogenic, and anti-fibrotic effects. Achieving sustained release is one of the important reasons for the recognition of hydrogels. In a rat model of Asherman syndrome, Liu et al. loaded a cross-linked hyaluronic acid gel with MSC secretome to create a sustained release system that repaired endometrial damage and enabled viable pregnancy (Liu et al., 2019). Hydrogels composed of bovine collagen type I (COLI) and low molecular weight hyaluronic acid (LMWHA) or COLI and PEG could control the release of active AD-MSC secretome, counteracting 6-OHDA toxicity while upregulating antioxidant enzymes Sirtuin 3 (SIRT3) in a neurodegeneration-related experimental setting (Chierchia et al., 2020). Liguori et al. proposed that trophic factors have different release kinetics and hydrogelation by a study of bioactive decellularized cardiac extracellular matrix-based hydrogels to release MSC secretome-derived trophic factors in a sustained manner, which is related to the initial concentration of conditioned medium (CM) in the gel (Liguori et al., 2021).

Pro-regeneration is the main function of the MSC secretome, and encapsulation with hydrogels significantly enhances the therapeutic effect, especially in skin lesions. In diabetic wounds mimicking fibroblasts cultured in a high-glucose environment, cell-free hydrogel dressings loaded with MSC-CM were shown to improve cell proliferation and migration through controlled release of bioactive factors (Sears et al., 2021). Pro-regenerative and pro-angiogenic can promote rapid skin repair. Kraskiewicz et al. demonstrated that collagen hydrogel maintained the therapeutic effect of AD-MSC secretome and improved chronic wound healing through proliferation of skin-derived cells and increased angiogenesis of endothelial cells (Kraskiewicz et al., 2021). Similarly, MSC-CM/hydrogel limited inflammatory development, promoted re-epithelialization and granulation tissue formation, and attenuated fibrotic and hypertrophic scar tissue formation outside the wound in third-degree burns in mice (Zhou et al., 2019a).

### 3.1 Hydrogels Encapsulate Mesenchymal Stem Cells-Extracellular vesicles

Hydrogel encapsulation can help to achieve spatio-temporal control of MSC-EVs activity *in vivo*, thereby increasing the therapeutic efficiency of EVs in various disease studies. In mice with renal ischemia-reperfusion injury, EVs released from matrix metalloproteinase-2-sensitive self-assembled peptide (KMP2) hydrogels demonstrated similar structures and bioactivities to fresh, isolated EVs, indicating that the hydrogels can effectively preserve biological function of EVs. The experimental results also proved that KMP2-EVs better promote endothelial cell proliferation and angiogenesis, and subsequently reduce renal chronic renal fibrosis, compared to KMP2 or EVs alone (Zhou et al., 2019b). *In vivo* tracking of labeled EVs showed that RGD peptide hydrogel increased EV retention and stability and promoted the efficacy of MSC-derived let-7a-5p-containing EVs in the treatment of AKI models (Zhang et al., 2020b). PEG hydrogels were found to sustain the release of EVs for up to 1 month in a rat model of chronic liver fibrosis (Mardpour et al., 2019). In the study of skin aging treatment, after subcutaneous injection treatment, CS hydrogel also prolonged the release of EVs and significantly increased the retention of EVs *in vivo*, delaying the skin-aging process (Zhao et al., 2021). In addition, hydrogel encapsulation also provides a new method of delivery of EVs compared to traditional arterial, intravenous, or intramuscular injection. For example, EVs were physically encapsulated in a GelMA hydrogel network covering the surface of the heart, and this local delivery could significantly improve retention (Tang et al., 2021).

### 3.2 Hydrogels Encapsulate Mesenchymal Stem Cells-Derived Exosomes

According to the diameter of the vesicles, EVs can be further divided into micro-vesicles (200–1,000 nm), exosomes (30–150 nm) and apoptotic bodies (800–5,000 nm). This subset of exosomes has been recognized as a new candidate for cell-free therapy of various diseases. Like MSC-EVs, the hydrogel enhanced the retention and stability of MSC-Exos *in vivo*. Yang et al. discovered that the thermosensitive PF-127 hydrogel delivered UC-MSC-exos, enhanced the regenerative capacity of exosomes, and promoted diabetes compared with UC-MSC-exos, UC-MSC-exos, PF-127-only, or control treatments (Yang et al., 2020b). Li et al. grafted MSC-Exos immobilized in peptide-modified adhesive hydrogels. The efficient retention and sustained release of MSC-Exos could attenuate inflammation and oxidation resulting in significant neurological recovery and urinary tissue protection, thereby effectively treating SCI (Li et al., 2020). In mice with hind limb ischemia, Gaussia luciferase imaging verified that CS hydrogels significantly increased the stability of proteins and microRNAs in MSC-Exos, and further enhanced MSC-Exos endothelial protection and pro-angiogenic capacity (Zhang et al., 2018).

### 3.3 Hydrogels Encapsulate Mesenchymal Stem Cells-Small Extracellular Vesicles

It is difficult to purify already pure exosome subsets further so people usually purify vesicles with a diameter of less than 200 nm, so people refer to them as small extracellular vesicles (sEVs). Yang et al. suggested that intra-articular injection of Diels-Alder cross-linked hyaluronic acid/PEG hydrogels achieved sustained release of MSC-sEVs through degradation control, enhancing the efficacy of MSC-sEVs in improving OA (Yang et al., 2021). In a model of MI, the addition of alginate hydrogels to MSC-sEVs significantly increased their retention in the heart, decreased cardiomyocyte apoptosis, and increased scar thickness and angiogenesis compared with sEV-only treatment (Lv et al., 2019).

## 4 Conclusion and Future Perspectives

In conclusion, the clinical translation of hydrogel encapsulation into MSCs and their secretome is a novel step to a very promising technology. Biomaterials are now a research hotspot, garnering more attention among researchers in the medical sciences. As a representative, hydrogel is summarized in this paper as a carrier to encapsulate MSCs and the related literature on their secretome is reviewed. At present, although there are many preclinical studies on hydrogel-encapsulated MSCs, the research is not deep enough, and clinical research therein remains sparse. There are still many gaps in the understanding of targeting of MSCs *in vivo*. The current research on hydrogel encapsulation tends towards cell-free therapy, and the MSC secretome may be the future development direction. However, MSCs still play an irreplaceable role in treatment of utilizing MSC differentiation. The texture of the gel is soft, and most of them are currently suitable for soft tissue, which limits the application on hard tissue to a certain extent. For the delivery of hard tissue MSCs and their secretome, more materials need to be explored to supplement. The composition of most gel carriers with obvious functions is relatively complex, and it may be possible to undertake further study in the future to achieve a simpler composition with stronger functions.
